# Excessively prolonged PR interval in a patient with worsening shortness of breath: a case report

**DOI:** 10.1186/s12872-025-04512-2

**Published:** 2025-02-17

**Authors:** Yanjuan Zhang, Zhenyang Jiang, Mingfang Li

**Affiliations:** 1 Department of Cardiology, the First Affiliated Hospital of Nanjing Medical University, Nanjing, China; 2 Department of Cardiology, the Affiliated Suqian First People’s Hospital of Nanjing Medical University, Suqian, China

## Abstract

**Background:**

Excessive prolongation of the PR interval indicates the potential for atrioventricular (AV) asynchrony, resulting in severe impairment of cardiac function.

Case presentation.

A 72-year-old man presented to the cardiology department with a history of worsening shortness of breath and chest tightness over the past 3 years. The electrocardiogram (ECG) showed sinus rhythm with a prolonged PR interval of 400 ms. The echocardiogram revealed mild mitral valve regurgitation with mitral E-A fusion during ventricular diastole. The patient received left bundle branch area pacing to shorten the AV conduction time.

**Conclusion:**

In patients with symptomatic AV block, reflected by an excessively prolonged PR interval, prompt decision-making regarding cardiac pacing therapy can help relieve clinical symptoms and enhance the patient's quality of life.

## Introduction

PR prolongation, also known as first-degree atrioventricular (AV) block, is a clinical condition characterized by delayed AV conduction between the atrium and the ventricle, with the AV node being the most commonly affected site[1]. PR prolongation is defined by a PR interval greater than 200 ms on the electrocardiogram (ECG). Patients with PR prolongation are generally asymptomatic and do not have significant complications. Treatment is usually not necessary. However, when the PR interval extends to more than 300 ms, or even 350 ms, the first-degree AV block is referred to as “marked” or “excessive”.

The excessive prolongation of the PR interval has received increased attention in recent years[1], as it indicates the potential for AV asynchrony, resulting in severe impairment of cardiac function. We present a case of a patient who has an excessively prolonged PR interval and is experiencing worsening shortness of breath.

## Case presentation

**Fig. 1:**
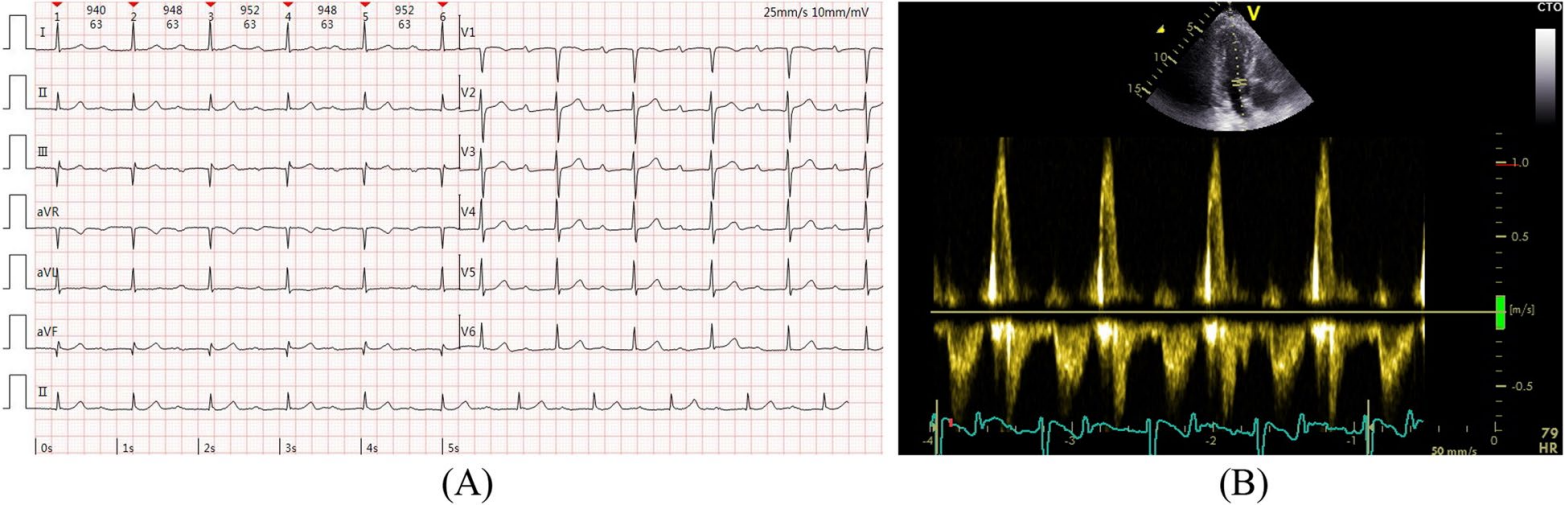
**A** Excessively prolonged PR interval on ECG before pacemaker implantation. **B** E and A wave fusion on echocardiogram before pacemaker implantation

A 72-year-old man presented to the cardiology department with a history of worsening shortness of breath and chest tightness over the past 3 years. He had no history of smoking, hypertension, diabetes mellitus, or cardiovascular diseases. During his physical examination, his blood pressure was measured at 108/71 mmHg, and his heart rate was 68 bpm. The distance walked in the 6-min walk test (6MWT) was 300 m. His ECG showed sinus rhythm with a prolonged PR interval of 400 ms (Fig. 1A). His echocardiogram revealed the fusion of the E and A waves with the duration of the E and A peak shortened to 200–250 ms in the mitral valve flow spectrum (Fig. 1B) and mild mitral valve regurgitation during ventricular diastole. His left ventricular (LV) systolic function was found to be normal, with a left ventricular diastolic diameter (LVDd) of 46 mm, a left ventricular ejection fraction (LVEF) of 64%, and a left ventricular global longitudinal strain (LV-GLS) of −17.3%. His level of NT-proBNP was mildly elevated (152.9 pg/ml, with a normal range of 0–125 pg/ml). Coronary artery CTA suggested 50% stenosis in the right coronary artery (RCA) and 55% stenosis in the left anterior descending artery (LAD), while results of laboratory tests were all normal.

**Fig. 2:**
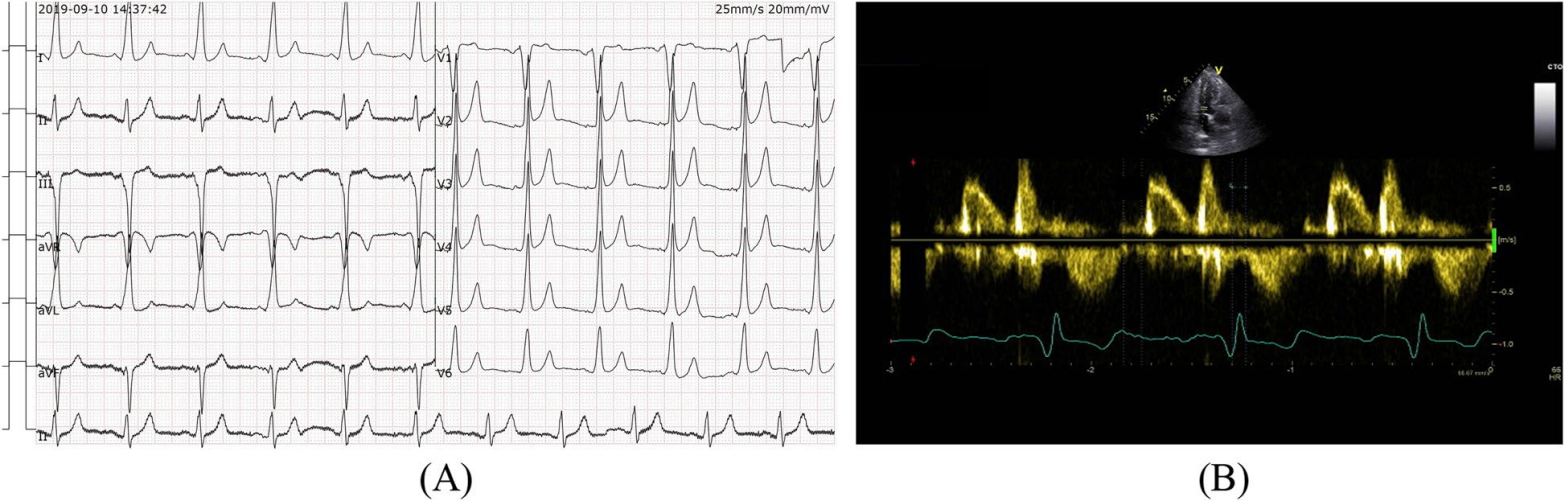
**A** Normal PR interval on ECG after pacemaker implantation. **B** Normal E and A wave on echocardiography after pacemaker implantation

Decision-making: The symptoms of this patient could be attributed to the impaired cardiac function caused by the delayed conduction in the AV node, reflected by an excessively prolonged PR interval. Delayed conduction in the AV node significantly reduces the effective filling period of the ventricular diastolic phase, leading to cardiac dysfunction. To address this, the patient received left bundle branch area pacing to shorten the atrioventricular conduction time. After pacemaker implantation, his PR interval was reduced to 120 ms (Fig. 2A), and his E and A duration returned to be in the normal range on the echocardiogram (Fig. 2B). Four months later, his symptoms were remarkably relieved, and the distance walked in the 6MWT was 600 m. During the 5-year follow-up, he remained clinically stable.

## Discussion

In a normal heart, sinus node depolarization propagates through the right and left atrial myocytes, reaching the AV node within 200 ms[2]. The PR interval commonly falls within the range of 120–200 ms, with the upper limit time of 220 ms[3]. The prevalence of PR prolongation is less than 1% in individuals aged < 60 years, but it increases to 6% in those ≥ 60 years old[1]. The prevalence of a PR interval ≥ 300 ms is extremely low, estimated to be less than 1 in 10,000 cases. For a PR interval exceeding 350 ms, the prevalence is even lower, and there is currently no available data on this specific condition[3]. Individuals with an excessively prolonged PR interval have an elevated risk of atrial fibrillation, heart failure, and all-cause mortality[4–6].

**Fig. 3:**
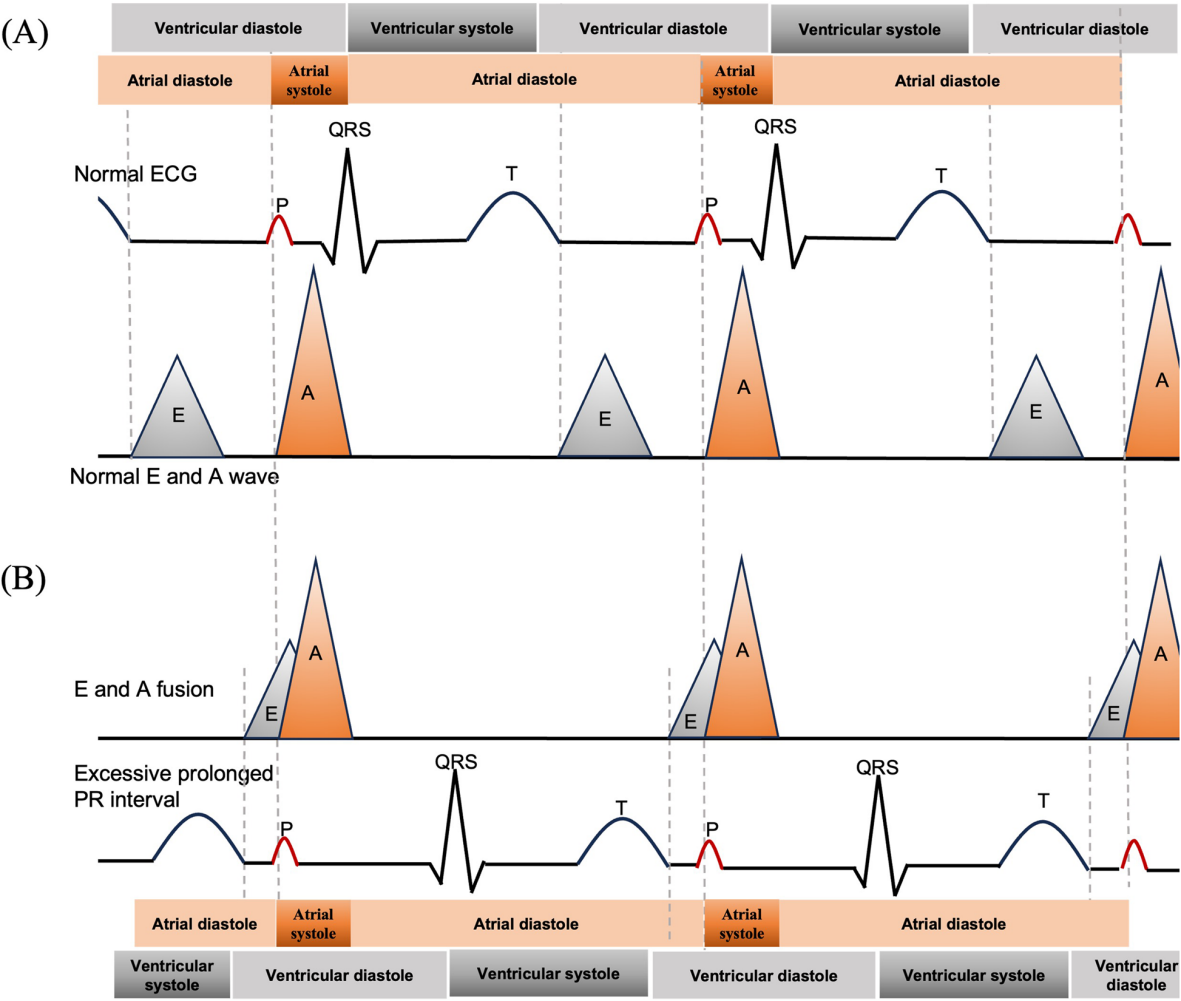
Illustration of mitral valve flow spectrum with a normal PR interval (**A**) and excessively prolonged PR interval (**B**)

Atrial depolarization (the P wave) typically occurs near the end of diastole in a normal heart. The excessive AV delay (i.e., prolonged PR interval) causes atrial depolarization to occur near the beginning of diastole (i.e., after the end of the T wave). In the mitral valve flow spectrum on the echocardiogram, the E wave is formed during rapid filling diastolic period, and the A wave represents atrial contraction. The duration of the E and A peaks corresponds to the effective filling diastolic period of the left ventricle throughout the cardiac cycle, typically ranging from 300 to 400 ms (Fig. 3A). The excessive AV delay leads to the fusion of the E and A waves, as the E wave is delayed and overlaps with the A wave. The shortened duration of this fused E and A peaks means that the effective filling diastolic period of the left ventricle is shortened (Fig. 3B). It will subsequently lead to a decrease in the stroke volume during left ventricular systole. Meanwhile, the hindered ventricular filling due to significantly delayed AV conduction may contribute to mitral regurgitation during ventricular diastole. The plausible mechanism involves early atrial contraction during diastole, with atrial relaxation starting before ventricular systole. This alteration affects the atrial-ventricular pressure gradient, increasing the likelihood of mitral regurgitation and exacerbating conditions such as heart failure. Taken together, cardiac function will be impaired in cases with an excessively prolonged PR interval.

An excessively prolonged PR interval is found to be associated with the subsequent need for permanent pacing in patients with symptoms attributable to AV block [7]. Small uncontrolled trials have suggested some symptomatic and functional improvement by pacing patients with PR intervals greater than 300 ms by decreasing the time for AV conduction [8]. For these patients with symptoms that are clearly attributable to the AV block, permanent pacemaker implantation is reasonable, supported by Class IIa recommendations in guidelines (Level of evidence: C) [9]. The typical pacing option is a dual-chamber pacemaker in these patients. It paces the right ventricle when there is a delay or failure in conduction from the atrium to the ventricle, ensuring that the ventricles contract appropriately. However, long-term RV pacing may cause ventricular dyssynchrony, worsening heart failure, and increasing the risk of arrhythmias. Newer pacing techniques like left bundle branch area pacing offer better synchronization and outcomes[10]. The largest European multicentre study (MELOS study) indicates left bundle branch area pacing is a feasible primary pacing technique for all-comers regardless of the pacing indication[11]. In this case, left bundle branch area pacing was successfully achieved, and the clinical symptoms relieved notably after his AV conduction time was reduced by cardiac pacing.

One limitation of this case study is that we did not perform an electrophysiology study to definitively determine the level of AV conduction delay. While we suspected that the delay was primarily in the AV node based on the patient's clinical presentation and echocardiographic findings, the possibility of infranodal conduction disease cannot be excluded. In future cases, electrophysiological evaluation should be considered to better define the conduction abnormality. The lack of BNP testing during the follow-up and the absence of an ergospirometric test are also limitations of our study.

## Conclusion

In patients with symptomatic AV block, reflected by an excessively prolonged PR interval, prompt decision-making regarding cardiac pacing therapy can help relieve clinical symptoms and enhance the patient’s quality of life.

## Data Availability

No datasets were generated or analysed during the current study.

## References

[1] Jackson LR 2nd, Ugowe F. Epidemiology and Outcomes Associated with PR Prolongation. Card Electrophysiol Clin. 2021;13(4):661–9. 10.1016/j.ccep.2021.06.007. 34689893 10.1016/j.ccep.2021.06.007 PMC9918374

[2] Nagueh SF, Smiseth OA, Appleton CP, et al. Recommendations for the Evaluation of Left Ventricular Diastolic Function by Echocardiography: An Update from the American Society of Echocardiography and the European Association of Cardiovascular Imaging. J Am Soc Echocardiogr. 2016;29(4):277–314. 10.1016/j.echo.2016.01.011. 27037982 10.1016/j.echo.2016.01.011

[3] Aro AL, Anttonen O, Kerola T, et al. Prognostic significance of prolonged PR interval in the general population. Eur Heart J. 2014;35(2):123–9. 10.1093/eurheartj/eht176. 23677846 10.1093/eurheartj/eht176

[4] Cheng S: Long-term Outcomes in Individuals With Prolonged PR Interval or First-Degree Atrioventricular Block. *Jama* 2009, 301(24). 10.1001/jama.2009.888 10.1001/jama.2009.888 PMC2765917 19549974

[5] Crisel RK, Farzaneh-Far R, Na B, et al. First-degree atrioventricular block is associated with heart failure and death in persons with stable coronary artery disease: data from the Heart and Soul Study. Eur Heart J. 2011;32(15):1875–80. 10.1093/eurheartj/ehr139. 21606074 10.1093/eurheartj/ehr139 PMC3202329

[6] Barold SS, Ilercil A, Leonelli F, et al. First-degree atrioventricular block. J Interv Card Electrophysiol. 2007;17(2):139–52. 10.1007/s10840-006-9065-x. 10.1007/s10840-006-9065-x 17334913

[7] Tracy CM, Epstein AE, Darbar D, et al. 2012 ACCF/AHA/HRS Focused Update of the 2008 Guidelines for Device-Based Therapy of Cardiac Rhythm Abnormalities. Circulation. 2012;126(14):1784–800. 10.1161/CIR.0b013e3182618569. 22965336 10.1161/CIR.0b013e3182618569

[8] Barold SS. Indications for permanent cardiac pacing in first-degree AV block: class I, II, or III? Pacing Clin Electrophysiol. 1996;19(5):747–51. 10.1111/j.1540-8159.1996.tb03355.x. 8734740 10.1111/j.1540-8159.1996.tb03355.x

[9] Epstein AE, DiMarco JP, Ellenbogen KA, et al. 2012 ACCF/AHA/HRS focused update incorporated into the ACCF/AHA/HRS 2008 guidelines for device-based therapy of cardiac rhythm abnormalities: a report of the American College of Cardiology Foundation/American Heart Association Task Force on Practice Guidelines and the Heart Rhythm Society. Circulation. 2013;127(3):e283-352. 10.1161/CIR.0b013e318276ce9b. 23255456 10.1161/CIR.0b013e318276ce9b

[10] Wang Y, Zhu H, Hou X, et al. Randomized Trial of Left Bundle Branch vs Biventricular Pacing for Cardiac Resynchronization Therapy. J Am Coll Cardiol. 2022;80(13):1205–16. 10.1016/j.jacc.2022.07.019. 36137670 10.1016/j.jacc.2022.07.019

[11] Jastrzebski M, Kielbasa G, Cano O, et al. Left bundle branch area pacing outcomes: the multicentre European MELOS study. Eur Heart J. 2022;43(40):4161–73. 10.1093/eurheartj/ehac445. 35979843 10.1093/eurheartj/ehac445 PMC9584750

